# Correction: Winkler et al. The Adhesion G-Protein-Coupled Receptor GPR115/*ADGRF4* Regulates Epidermal Differentiation and Associates with Cytoskeletal KRT1. *Cells* 2022, *11*, 3151

**DOI:** 10.3390/cells12131677

**Published:** 2023-06-21

**Authors:** Romy Winkler, Marianne Quaas, Stefan Glasmacher, Uwe Wolfrum, Torsten Thalheim, Jörg Galle, Knut Krohn, Thomas M. Magin, Gabriela Aust

**Affiliations:** 1 Research Laboratories and Clinic of Orthopedic Surgery, Traumatology and Plastic Surgery, Leipzig University and University Hospital, 04103 Leipzig, Germany; 2 Research Laboratories and Clinic of Visceral, Transplantation, Thoracic, and Vascular Surgery, Leipzig University and University Hospital, 04103 Leipzig, Germany; 3 Institute of Molecular Physiology, Molecular Cell Biology, Johannes Gutenberg University of Mainz, 55128 Mainz, Germany; 4 Interdisciplinary Center for Bioinformatics (IZBI), Leipzig University, 04107 Leipzig, Germany; 5 Core Unit DNA-Technologies, Leipzig University, 04103 Leipzig, Germany; 6 Division of Cell and Developmental Biology, Institute of Biology, Leipzig University, 04103 Leipzig, Germany

In the original publication [1], the figures were not sharp enough. The enhanced Figure 1, Figure 2, Figure 3, Figure 4, Figure 5 and Figure 6 appear below. The figures, prepared by the authors, had high quality from the very beginning. 

The authors state that the scientific conclusions are unaffected. This correction was approved by the Academic Editor. The original publication has also been updated.

## Figures and Tables

**Figure 1:**
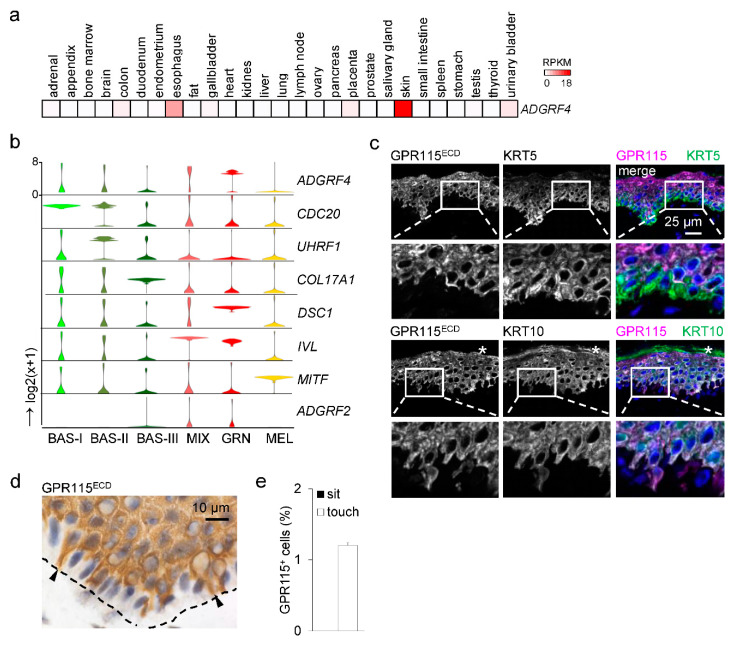
**GPR115/*ADGRF4* is present in rare basal and almost all suprabasal keratinocytes.** (**a**) Transcriptomic profile of *ADGRF4* in human tissues (bulk RNAseq) [11], given as RPKM (reads per kilobase per million mapped reads). (**b**) Reanalysis of scRNAseq data of human epidermis [1]. Violin plots of relative gene expression from basal cell types I-III (BAS), a mixed cluster (MIX, characterizing the transition from type-IV basal cell to spinous and granular keratinocytes), granular keratinocytes (GRN), and melanocytes (MEL). (**c**) Costaining for GPR115 and keratins of normal skin cryosections (star: cornified layer). (**d**) In images of a horse radish peroxidase based immunostained epidermis, few GPR115^+^ basal cells were seen at higher magnification (arrows; broken line: basal membrane). (**e**) Quantitation of GPR115^+^ cells in the basal layer, which either “sit at” or “touch” the basal membrane with extensions (dorsal skin, n = 4 donors; n = 1000–2000 basal cells/donor, mean ± SEM).

**Figure 2:**
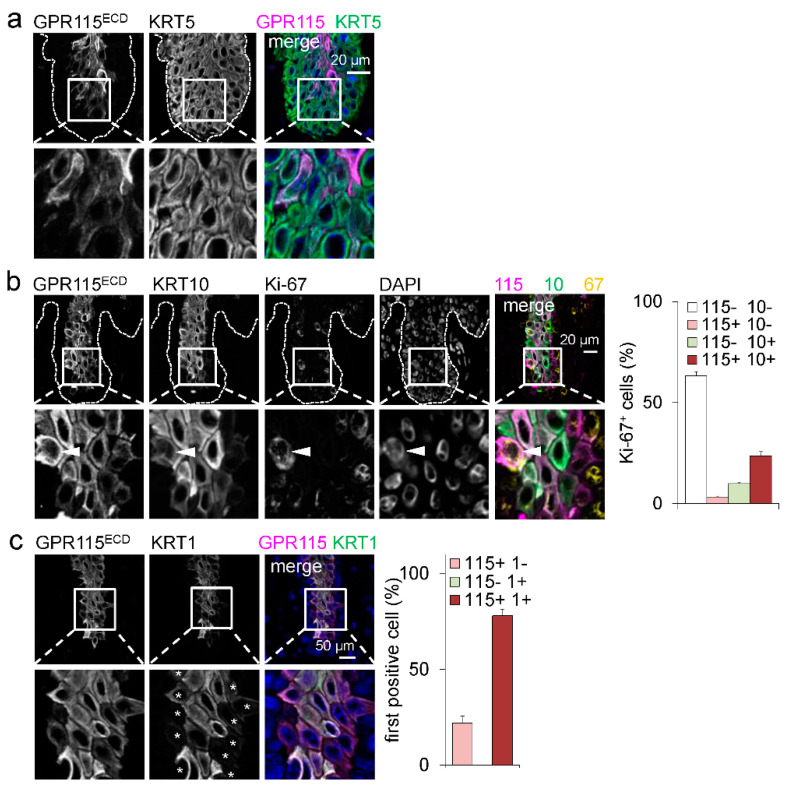
**GPR115 and KRT1/10 were delayed in rete ridges of lesional psoriatic skin.** (**a**) In a rete ridge, suprabasal keratinocytes still expressed KRT5, whereas the emergence of GPR115^+^ keratinocytes was delayed. (**b**) Triple immunostaining of Ki-67, GPR115, and KRT10 revealed a markedly increased proportion of proliferating Ki-67^+^ in the suprabasal layer. Some of these Ki-67^+^ cells expressed GPR115 (arrowhead). The epidermal basal membrane is indicated by a broken line. Right: Quantitation of the percentage of GPR115^+^ cells among Ki-67^+^ suprabasal keratinocytes (n = 10 optical fields, means ± SEM). (**c**) Determination of whether the first stained suprabasal cell is GPR115^+^ and/or KRT1^+^. Left: Costaining; in the insert the rated cells are indicated by asterisks. Right: quantitation of the first GPR115- or KRT1-Ab-stained cells (n = 10 optical fields, means ± SEM).

**Figure 3:**
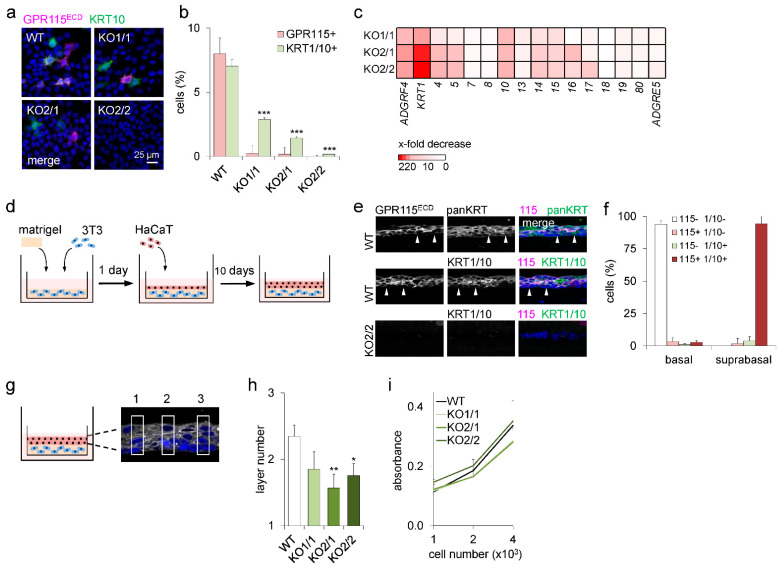
**Loss of *ADGRF4* abrogated *KRT1* and disturbed differentiation in HaCaT keratinocytes.** (**a**) Monolayered HaCaT WT cells and GPR115KO clones were costained for GPR115 and KRT10. (**b**) The percentages of GPR115^+^ and KRT10^+^ cells were determined in each clone (n = 10 optical fields, n = 70–100 cells/field, means ± SEM, *** *p* < 0.001 compared with WT). (**c**) RNA sequencing of HaCaT WT and GPR115KO clones. The ratio of FKPM values in clones and WT is given as an x-fold decrease. (**d**) Scheme generating organotypic skin constructs consisting of Matrigel-embedded 3T3 fibroblasts overlayered with HaCaT WT or GPR115KO cells. (**e**) Cross-cryosections of constructs built with HaCaT WT cells (upper panels) and the GPR115KO2/2 clone (lower panel) were costained for GPR115 and keratins. (**f**) The percentages of GPR115^+^ and KRT1/10^+^ cells were determined in these constructs built with HaCaT WT cells (n = 10 optical fields, n = 20–27 cells/field, means ± SEM). (**g**) Scheme of the determination of the HaCaT layer number. The number of DAPI^+^ nuclei was counted in 10 uniform segments; the distances between the segments were equal (three segments are shown). (**h**) Quantitation of the HaCaT layer number in organotypic skin constructs (n = 5–8 experiments/WT or GPR115KO clone, 3 cross sections/experiment, 10 segments/cross section, means ± SEM, * *p* < 0.05, ** *p* < 0.01 compared with WT). (**i**) Crystal violet assay of 1 × 10^3^, 2 × 10^3^, and 4 × 10^3^ seeded HaCaT WT and GPR115KO cells. Attached cells over time were quantified. Absorbance was measured after 48 h at 590 nm (n = 3 experiments, n = 3 replicates/experiment; means ± SEM).

**Figure 4:**
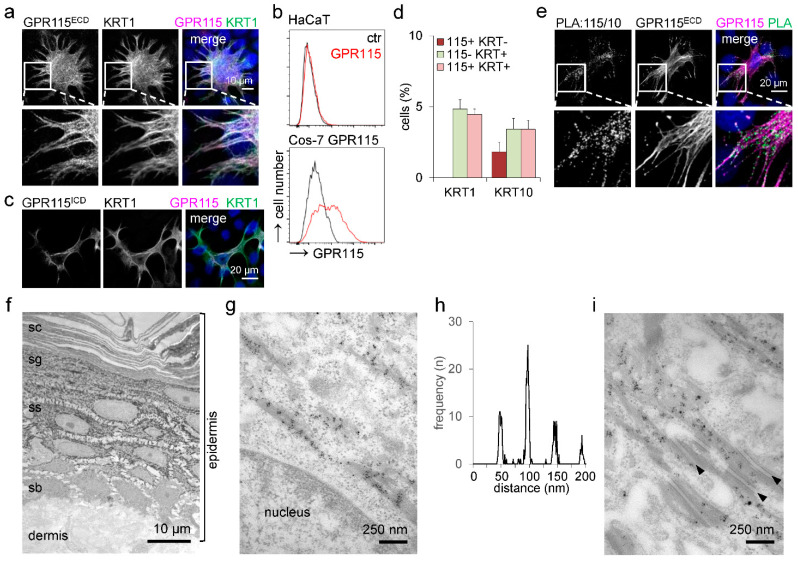
**Endogenous GPR115 colocalizes with KRT1/10.** (**a**) GPR115 and KRT1 costaining of HaCaT cells. The insert illustrates the obvious colocalization and the filament-like, partly dotted GPR115 staining. (**b**) HaCaT WT cells and Cos-7 cells, transfected with GPR115 pcDNA3.1, were cell-surface-stained with the GPR115^ECD^ Ab and analyzed by flow cytometry. (**c**) GPR115^ICD^ and KRT1 Ab costaining of HaCaT cells. (**d**) Calculation of the percentage of GPR115^ECD^-labeled HaCaT cells costained with the KRT1 or KRT10 Abs (n = 10 optical fields, n = 220–320 cells/field, means ± SEM). (**e**) Proximity ligation assay (PLA) applying the rabbit GPR115^ECD^ and mouse KRT10 Abs in HaCaT WT cells. After visualization of the PLA interaction dots, the cells were stained with a fluorophore-labeled antirabbit secondary Ab to visualize GPR115. (**f**–i) GPR115^ECD^ Ab immunogold labeling of normal skin via electron microscopy. (**f**) Immunogold particles were present in all suprabasal, nonkeratinized epidermal layers (sb: stratum basale, ss: stratum spinosum, sg: stratum granulosum, sc: stratum corneum). (**g**) The immunogold particles were located at keratin filaments and grouped with defined distances from each other. (**h**) These distances were quantified in pictures; the frequencies of a certain distance between the particle groups are given. The distances between groups were multiples of 48 nm (48.8 ± 0.4, 96.2 ± 0.3, 144.8 ± 0.4, 193.1 ± 0.5 nm; n = 16 figures, 10–45 particle groups at filaments/figure, means ± SEM). (**i**) Alongside desmosomes (arrowheads), immunogold particle groups were also located at filaments. The distance of such groups to the desmosomes was 57.6 ± 1.3 nm (n = 8 pictures, 2–6 particle groups/picture, means ± SEM).

**Figure 5:**
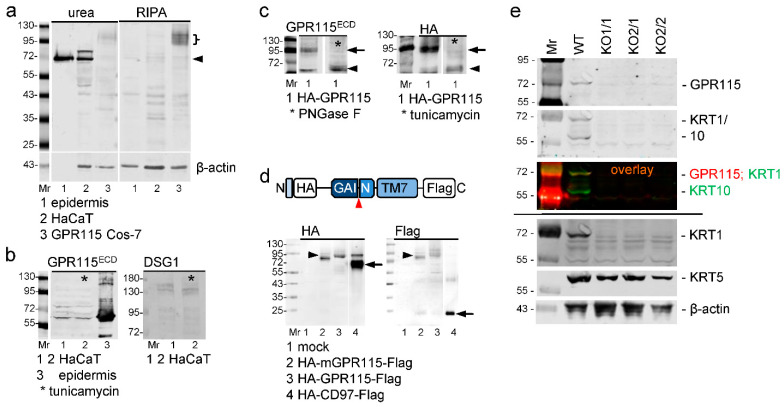
**Endogenous GPR115 is not N-glycosylated and is likely not cleaved at the GPS.** (**a**) Western blot analysis of epidermis, HaCaT, and HA-GPR115 Cos-7 cells in urea-based (**left**) and detergent-based (**right**) lysates applying the GPR115^ECD^ Ab. Only 1 μg of epidermal protein/lane was blotted, while 40 μg/lane of the other lysates were blotted. In the lower part, the loading control using a β-actin Ab is shown. It is negative for the epidermis because only 1 μg of protein was applied. (**b**) Western blot analysis of lysates (urea buffer) of epidermis and HaCaT cells; two different blots are shown. Tunicamycin (*) did not decrease the molecular weight of endogenous GPR115 (**left**) but decreased that of desmoglein 1 (DSG1, **right**), which served as a positive control. (**c**) Western blot analysis of lysates of HA-GPR115 Cos-7 cells treated with PNGase F (**left**) or of HA-GPR115 Cos-7 cells cultured with tunicamycin (**right**); the Abs used are indicated. Most transfected GPR115 is N-glycosylated (arrows); deglycosylation reduced the molecular weight to 65 kDA (arrowheads). (**d**) Lysates of Cos-7 transfected with constructs encoding N-terminal HA- and C-terminal Flag-tagged mouse or human GPR115 and human CD97 were analyzed with the indicated Abs by Western blot; the coding part of the constructs is shown schematically above (arrowhead: putative GPS). In the blots, arrowheads indicate uncleaved full-length human GPR115, and the arrows indicate the N- and C-terminal fragments of cleaved CD97 (positive control). Mouse GPR115, a little smaller than human GPR115 also was not cleaved. (**e**) Western blot analysis of urea-based lysates of HaCaT WT cells and the various GPR115 clones. The applied Abs are indicated on the right. Upper part: First, the blot was consecutively incubated with the rabbit GPR115^ECD^ and mouse KRT1/10 primary Abs. Afterwards both fluorophore-labeled secondary Abs were applied; pictures taken from this blot are shown (the single Abs in grey and their overlay colored). Lower part: same blot after stripping and restaining; the stripped blot was horizontally cut, and the three parts were incubated with either the KRT1, KRT5, or β-actin Abs.

**Figure 6:**
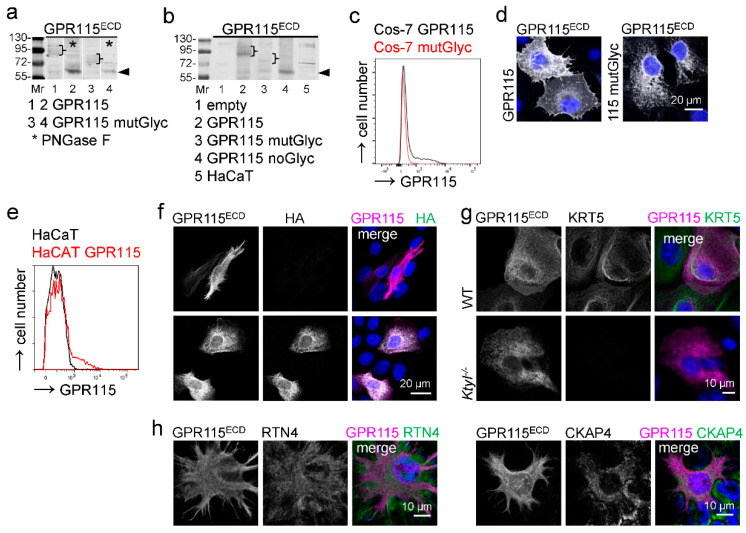
**GPR115 transfected into Cos-7 cells is not keratin-associated.** (**a**) Western blot analysis of Cos-7 cells transfected with the various indicated GPR115 pcDNA3.1 constructs. Mutation of seven potential GPR115 N-glycosylation sites (GPR115 mutGlyc) reduced the molecular weight of transfected GPR115 from ~90–110 to ~75–85 kDa (brackets). PNGase F treatment (*) of the cellular lysates resulted in a ~65 kDa band (arrowhead); thus, further potential GPR115 N-glycosylation sites were used. (**b**) Consistently, mutation of all potential GPR115 N-glycosylation sites (GPR115 noGlyc) reduced the molecular weight to ~63–65 kDa (arrowhead). (**c**,**d**) Cos-7 cells, transfected with GPR115 or GPR115 mutGlyc pcDNA3.1, were stained for GPR115. Mutant GPR115 mainly disappeared from the cell surface, as seen in flow cytometry (**c**) and in stained monolayered cells (**d**). (**e**–**g**) HaCaT WT cells were transfected with HA-GPR115 pcDNA3.1. (**e**) These cells were stained with the GPR115^ECD^ Ab and compared with nontransfected cells in flow cytometry. (**f**) The transfected HaCaT cells were costained using the GPR115^ECD^ and HA Abs to differentiate between endogenous (HA-) and transfected (HA+) GPR115. (**g**) Mouse WT and keratin type I-deficient (*KtyI^-/-^*) keratinocytes were transfected with HA-GPR115 pcDNA3.1 and costained for KRT5 and GPR115; the GPR115^ECD^ Ab labeled only human GPR115. The staining pattern of transfected GPR115 was similar in WT and *KtyI^-/-^* keratinocytes. (**h**) HaCaT WT cells were costained for GPR115 with the ER tubule marker RTN4 and the ER sheet marker CKAP4.
