# Supplementary material for: The Adhesion G-Protein-Coupled Receptor GPR115/ADGRF4 Regulates Epidermal Differentiation and Associates with Cytoskeletal KRT1
Source: Cells. 2022 Oct 7;11(19):3151. doi: 10.3390/cells11193151 (PMC9563031; doi:10.3390/cells11193151)
Supplement: Supplementary file 1 [file cells-11-03151-s001.zip › cells-1919427-SM.pdf]

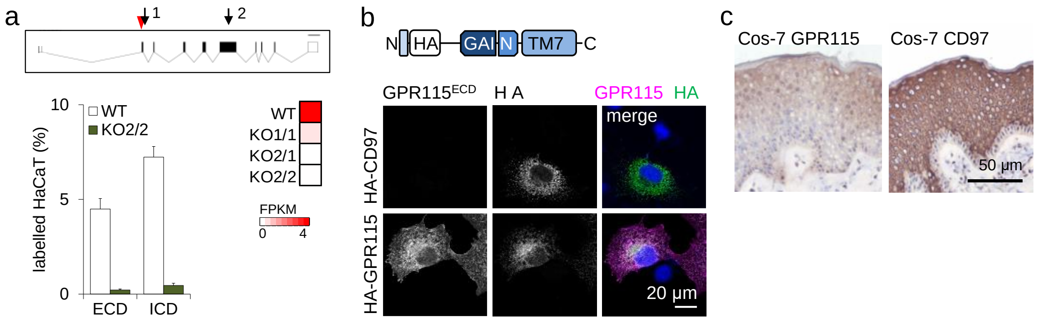

Figure S1. Validation of GPR115 Abs. To rigorously control Ab specificity, we carefully evaluated GPR115 Abs by using *ADGRF4*-deficient HaCaT keratinocytes and Cos-7 cells expressing tagged GPR115. By staining monolayered HaCaT keratinocytes, we identified two Abs (**Table S1**), one binding the ECD (GPR115^{ECD}) and the other near the C-terminus (GPR115^{ICD}), to stain ~4-8 % of the HaCaT wild-type (WT) cells (**Figure S1a**). Applying a CRISPR/Cas9 approach we generated three HaCaT GPR115 knock-out (KO) clones. Their *ADGRF4* level was quantified by RNAseq. Staining the HaCaT GPR115KO2/2 clone with the lowest *ADGRF4* level revealed that less than 0.5% of these cells still expressed GPR115, confirming Ab specificity. Afterwards, we validated Ab specificity using Cos-7 cells expressing tagged GPR115 or tagged CD97, used as a control (**Figure S1b**). One-to-one overlapping of the target with GPR115^{ECD} Ab-labeling was seen only with HA-GPR115 Cos-7 cells. Staining of these cells with the GPR115^{ICD} Ab was faint (not shown). Thus, we used the GPR115^{ECD} Ab for further analyses. To further confirm GPR115^{ECD} Ab specificity, we applied this Ab pre-absorbed with Cos-7 expressing GPR115 (**Figure S1c**).

(**a**) Validation of GPR115 Abs using HaCaT (WT) cells and GPR115KO clones. Upper part: Visualization of the present *ADGRF4* transcript (ENST00000283303). The black boxes represent the transcribed exons; arrows: binding sites of *ADGRF4* sgRNA1 (GPR115KO1 clone) and 2 (KO2); arrowhead: translation start site; scale bar 100 bp. Left: Percentage of GPR115$^+$ HaCaT WT or GPR115KO2/2 cells stained with the GPR115^{ECD} or GPR115^{ICD} Ab (n = 10 optical fields, n= 220-320 cells/field, mean ± SEM). Right: RNA-sequencing of HaCaT WT cells and GPR115KO clones; abundance of the *ADGRF4* transcript is given as FPKM (fragments per kilobase per million mapped reads).

(**b**) Validation of GPR115 Abs using tagged GPR115. Cos-7 cells were transfected either with HA-tagged GPR115 pcDNA3.1 (the encoded part of this construct is shown schematically above), or with HA-tagged CD97 pcDNA3.1 as a control and co-stained using the GPR115^{ECD} and HA Abs. All GPR115$^+$ cells were HA$^+$. Non-transfected cells (HA$^-$) were GPR115$^-$. HA-CD97$^+$ Cos-7 cells were GPR115$^-$, confirming Ab specificity.

(**c**) Validation of the GPR115^{ECD} Ab, preabsorbed with GPR115-expressing Cos-7 cells**.** For this reason, Cos-7 cells were transfected with GPR115 or CD97 pcDNA3.1. After 48 h, the detached and pelleted Cos-7 cells were incubated with the diluted GPR115^{ECD} Ab for 30 min while shaking. Afterwards, the cells were centrifuged and the supernatant, that is the pre-absorbed Ab, was used for immunostaining skin cryosections. Staining of suprabasal keratinocytes was markedly diminished when applying the Ab pre-absorbed with GPR115$^+$, but not with CD97$^+$ Cos-7 cells.

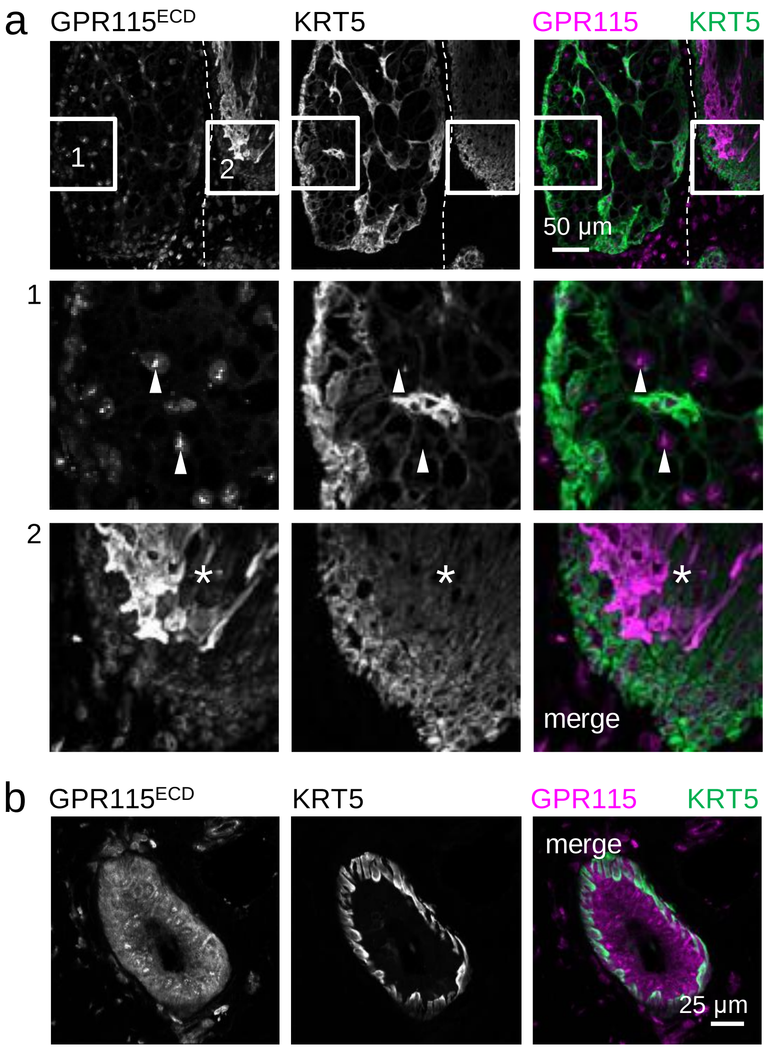

Figure S2. GPR115 is present in skin appendages. Consistent with epidermal labelling, GPR115 was present in epithelium-derived skin appendages such as the suprabasal cells of the outer hair root sheath and the secretory part of eccrine sweat glands. This expression profile is very similar to KRT1 and KRT10, but not to KRT5 (**Table S2**).

(**a, b**) Normal skin, dermis (cryosection), co-staining using the $GPR115^{ECD}$ and KRT5 Ab. (**a**) In a sebaceous gland (left in the figure, insert 1), only a few individual cells showed low GPR115 (arrowheads in insert 1), whereas KRT5-positive sebocytes lacked GPR115 staining. Hair follicles (right in the figure, insert 2), in analogy to interfollicular epidermis, showed GPR115 in suprabasal (asterisk in insert 2), but not basal $KRT5^{+}$ cells of the outer epithelial hair root sheath. (**b**) Secretory coil cells of a sweat gland expressed GPR115.

Table S1. Antibodies (Abs) for immunolabeling

Ab/antigen	source, mono (clone)/polyclonal	used for	reference/ company; catalog number
GPR115^{ECD}; PrEST, ECD (aa Q87-R180)	r, poly	IS, IH, WB	Merck (Darmstadt, Germany); HPA007131
GPR115^{ICD} #; ICD (aa S674-N693)	r, poly	IS	Affinity Biosciences (Shanghai, China); DF4943
KRT1	m, mono (HK1)	IS	Bio-Techne GmbH (Wiesbaden, Germany); NB100-2756
KRT1	gp, poly	WB	PROGEN Biotechnik GmbH (Heidelberg, Germany); GP-K1
KRT5	m, mono (XM26)	WB	BioTechne; NB120-17130
KRT10	m, mono (DE-K10)	IS, WB	PROGEN; 11414
CK1/10	m, mono (LH1)	IS, WB	Santa Cruz Biotechnology (Heidelberg, Germany); sc-53251
panKRT	m, mono (C11)	IS	Santa Cruz; sc-8018
desmoglein 1 (DSG1)	m, mono (B11)	WB	Santa Cruz; sc-137164
desmoplakin I+II	m, mono	IS	PROGEN, 65146
Ki-67	s, poly	IH	R&D systems; AF7617
β-actin	m, mono (AC-15)	WB	Merck; A5441
reticulon 4 (RTN4)	m, mono (C-4)	IS	Santa Cruz; sc-271878
cytoskeleton-associated protein 4 (CKAP4, CLIMP-63)	m, mono (G1/296)	IS	Enzo Life Sciences GmbH (Lörrach, Germany)
HA	m, mono (F-7)	IS	Santa Cruz; sc-7392
HA	r, mono (C29F4)	WB	Cell Signalling; 3724
FLAG	m, mono (9A3)	IS, WB	Cell Signalling; 8146
Alexa Fluor 488 anti-rabbit IgG; F(ab')$_2$	g, poly	IS, IH	Jackson ImmunoResearch Europe Ltd (Cambridgeshire, UK) 111-546-144
DyeLight 550 anti-rabbit IgG (H+L)	g, poly	IS, IH	Dianova GmbH (Hamburg, Germany); GtxRb-003-E550NHSX
Alexa Fluor 546 anti-mouse IgG; F(ab')$_2$	g, poly	IS, IH	Thermo Fisher; A-11018
Alexa Fluor 633 anti-mouse IgG; F(ab')$_2$	g, poly	IS, IH	Thermo Fisher; A21053
NorthernLights™ NL557 anti-sheep IgG	d, poly	IS, IH	Bio-Techne; NL010
Alexa Fluor488 anti-guinea pig IgG (H+L)	g, poly	IS, IH	Thermo Fisher; A-11073
ImmPress HRP, anti-rabbit	ho, polymer IgG	IH	Vector Laboratories (Newark, US); MP-7401
IRDye 680RD anti-rabbit IgG (H+L)	g, poly	WB	LI-COR Bioscience GmbH (Bad Homburg, Germany); 926-68071
IRDye 800CW anti-mouse IgG (H+L)	g, poly	WB	LI-COR; 926-32210
IRDye 800CW anti-guinea pig IgG (H+L)	d, poly	WB	LI-COR; 926-32411

aa amino acid, d donkey, ECD extracellular domain, g goat, gp guinea pig, h human, ho horse, ICD intracellular domain, IH immunohistology, IS immunostaining of monolayered cells, m mouse, PrEST precursor recombinant protein epitope signature tag, r rabbit, s sheep, WB western blot;
The GPR115^{ICD} Ab quality varied obviously between various batches, most are even useless. However, the company did not improve Ab quality until now.

Table S2. Expression profile of GPR115 and related keratins in normal skin (cryosections)

**anatomical structure**		**GPR115^{ECD1}**		**KRT1**		**KRT10**		**KRT5**	
		%	int	%	int	%	int	%	int
**epidermis**	stratum corneum	0	0	0	0	100	3	0	0
	stratum granulosum	100	3	100	3	100	2	0	0
	stratum spinosum	100	3	100	2	100	3	0	0
	stratum basale	0	0	0	0	0	0	100	3
**dermis**	scattered single or grouped cells*	*	2	0	0	0	0	0	0
**hair shaft**	cuticula cortex medulla	0	0	0	0	0	0	0	0
**hair follicle**	outer epithelial HRS, suprabasal	>66	3	<33	1	100	2	0	0
	outer epithelial HRS, basal	0	0	0	0	0	0	100	1
	inner epithelial HRS	0	0	0	0	0	0	0	0
**sebaceous glands**	basal sebocytes	0	0	0	0	100	3	100	2
	suprabasal sebocytes	0	0	0	0	100	2	0	0
**eccrine sweat glands: secretory part**	secretory cells	100	2	100	1	0	0	100	2
	myoepithelial cells	0	0	0	0	0	0	100	2
**eccrine sweat glands: acrosyringium**	basal cells	0	0	0	0	0	0	100	2
	luminal cells	0	0	0	0	100	3	0	0

percentage of stained cells (%): 0 none, < 33 %, 33-66 %, > 66 %, 100 % positive cells; staining intensity: 0 no, 1 weak, 2 moderate, 3 strong; *double immunostaining: most of scattered GPR115^{+} cells were CD45^{-}, CD31^{-}, S100β^{-}, but partly vimentin^{+}; HRS hair root sheath; int intensity

Table S3. Mutated N-glycosylation sites of GPR115

position	sequence	potential	jury	agreement result	mutated to	GPR115 mutGlyc	GPR115 noGlyc
61	NCSQ	0.6711	8/9	+	ACSQ	+	+
169	NIST	0.5508	5/9	+	AIST		+
177	NVTR	0.7406	9/9	++	AVTR	+	+
209	NASS	0.5249	5/9	+	AASS		+
229	NNSE	0.5933	6/9	+	ANSE		+
250	NTSE	0.6619	9/9	++	ATSE	+	+
257	NFSM	0.5653	7/9	+	AFSM		+
263	NNTT	0.4850	6/9	-	AATT		+
264	NTTE	0.6486	8/9	+	ATTE	+	+
286	NASQ	0.6029	6/9	+	AASQ		+
309	NVSL	0.7192	9/9	++	AVSL	+	+
340	NKTR	0.6022	9/9	++	AKTR	+	+
379	NYTS	0.6816	9/9	++	AYTS	+	+
679	NASL	0.4684	5/9	-	AASL		+
686	NGSK	0.6030	9/9	++	AGSK		+

Potential N-glycosylation sites within the GPR115 ECD were predicted by NetNGlyc 1.0.
